# Telomeres and Telomerase in the Development of Liver Cancer

**DOI:** 10.3390/cancers12082048

**Published:** 2020-07-24

**Authors:** Lena in der Stroth, Umesh Tharehalli, Cagatay Günes, André Lechel

**Affiliations:** 1 Department of Internal Medicine I, University Hospital Ulm, 89081 Ulm, Germany; lena.in@uni-ulm.de (L.i.d.S.); umeshtm.14@gmail.com (U.T.); 2 Department of Urology, University Hospital Ulm, 89081 Ulm, Germany; cagatay.guenes@uniklinik-ulm.de

**Keywords:** liver cancer, hepatocellular carcinoma, intrahepatic cholangiocarcinoma, telomere shortening, TERT promoter mutation, telomerase

## Abstract

Liver cancer is one of the most common cancer types worldwide and the fourth leading cause of cancer-related death. Liver carcinoma is distinguished by a high heterogeneity in pathogenesis, histopathology and biological behavior. Dysregulated signaling pathways and various gene mutations are frequent in hepatocellular carcinoma (HCC) and intrahepatic cholangiocarcinoma (iCCA), which represent the two most common types of liver tumors. Both tumor types are characterized by telomere shortening and reactivation of telomerase during carcinogenesis. Continuous cell proliferation, e.g., by oncogenic mutations, can cause extensive telomere shortening in the absence of sufficient telomerase activity, leading to dysfunctional telomeres and genome instability by breakage–fusion–bridge cycles, which induce senescence or apoptosis as a tumor suppressor mechanism. Telomerase reactivation is required to stabilize telomere functionality and for tumor cell survival, representing a genetic risk factor for the development of liver cirrhosis and liver carcinoma. Therefore, telomeres and telomerase could be useful targets in hepatocarcinogenesis. Here, we review similarities and differences between HCC and iCCA in telomere biology.

## 1. Introduction

Liver cancer is predicted to be the sixth most common tumor disease worldwide and the fourth leading cause of cancer-related death [1]. Liver cancer presents with a high heterogeneity in pathogenesis, histopathology and biological behavior. The heterogeneous disease in terms of etiologies reflects the poor prognosis of patients with liver cancer. The two most common types of liver cancer are hepatocellular carcinoma (HCC) (75–85% of cases) and intrahepatic cholangiocarcinoma (iCCA) (10–15% of cases) [1]. Most liver carcinomas are diagnosed at advanced stages despite the surveillance program of patients with liver cirrhosis to diagnose early liver tumors. To date, therapy options are limited to the multikinase inhibitors sorafenib and lenvatinib as first-line treatment options and regorafenib and cabozantinib as second-line treatment options for liver cancer patients [2,3,4]. A recent clinical trial revealed significantly longer overall and progression-free survival in patients with unresectable hepatocellular carcinoma, who received atezolizumab, a programmed death ligand 1 (PD-L1) inhibitor, combined with bevacizumab, a monoclonal antibody targeting the vascular endothelial growth factor (VEGF), in comparison to sorafenib only [5]. These findings point to new treatment options in patients with unresectable hepatocellular carcinoma and support the development of new therapy options.

The major risk factors in hepatocarcinogenesis are chronic viral infections with hepatitis B virus (HBV) and hepatitis C virus (HCV), heavy alcohol consumption, obesity, type 2 diabetes, smoking and long-term exposure to aflatoxin B [6]. Most liver tumors arise on the basis of chronic liver diseases and often result in liver fibrosis and cirrhosis formation, which itself represents a risk factor for tumor development. In western countries, HCC is mainly related to HCV, high alcohol consumption and non-alcoholic steatohepatitis (NASH) and connected with cirrhosis formation [7,8], whereas in Asia, most HCC patients are related to HBV infection; in addition, HCC can also develop in normal liver without fibrosis/cirrhosis or liver with limited fibrosis formation [9]. Interestingly, in Japan, chronic HCV infection is more common than HBV, and HCV infection accounts for the majority of HCC [10]. The burden of hepatocellular carcinoma is continuously growing due to increased rates of obesity, type 2 diabetes and nonalcoholic fatty liver disease (NAFLD), especially in low-risk HCC areas and thereby replaces viral- and alcohol-related chronic liver diseases [11]. Due to the diversity of risk factors, a high heterogeneity in liver tumors is reported. The molecular heterogeneity in terms of various gene mutations in liver cancer requires the identification of molecular targets for designing individualized therapies. Recent studies have described various sub-classification of HCC and iCCA tumor types [12,13,14]. Individual studies underline the importance of specific treatment options based on the tumor subtypes as a key to achieving a better overall survival of liver cancer patients [15,16,17]. The identification of dysregulated molecular pathways in premalignant lesions is required for an early disease detection in hepatocarcinogenesis [18]. A detailed description of molecular targets in the diversity of liver cancer subtypes would be beneficial for targeted therapies.

Telomere shortening and reactivation of telomerase, two common hallmarks of carcinogenesis, are described in a broad range of human cancers, including liver cancer [19,20,21]. Telomere shortening and reactivation of telomerase, through TERT promoter mutations, for example, represent genetic risk factors for the development of liver cirrhosis and liver cancer [12,22]. Therefore, disruptions in telomere biology could be a useful target for the treatment of liver cancer. In the following, we will present the current knowledge of telomere biology in HCC and iCCA.

## 2. Telomere Shortening in Liver Cirrhosis and Hepatocellular Carcinoma

Chronic liver disease is associated with chronic liver inflammation, which can lead to cell death and compensatory cell regeneration. The liver is characterized by a high regenerative reserve [23], which decreases in the context of chronic liver disease, consequently leading to telomere shortening and limiting the regenerative reserve of the liver. The frequent appearance of senescent hepatocytes in liver cirrhosis is the result of the loss of telomeric repeats and the extensive proliferation [21,24,25,26,27]. These senescent hepatocytes exhibit markers like p16^INK4a^ and p21^WAF1/Cip1^ and are positive for senescence-associated β-galactosidase staining. A disruption of the p53-signaling pathway overcomes the senescence checkpoint and leads to further cell division of hepatocytes with already-shortened telomeres until the telomeres become critically short. At this point, the cells enter the crisis checkpoint, which is characterized by massive cell death [25,27,28].

In the liver, telomerase activity is downregulated during early embryonic development, and telomerase activity is absent in the adult liver. The healthy liver is a slowly proliferating organ, and most hepatocytes are in a quiescent stage (only one out of 20,000 cells (0.005%) is in the cell cycle [29]). Thus, telomerase activity seems not to be essential for hepatocyte function in a healthy liver. However, telomere shortening occurs in the absence of sufficient telomerase activity in hepatocytes under conditions of chronic liver diseases or upon injury [21,25]. Importantly, low levels of telomerase activity were observed under regenerative conditions, indicating the potential physiological activation of telomerase in adult hepatocytes [30,31]. So far, two distinct mechanisms were made responsible for telomere shortening upon increased proliferative signals, either due to oncogene activation (e.g., *Ras* mutations or *c-Myc* amplification) or expression of viral oncogenic proteins: (i) in most of the cases, absence of sufficient telomerase reactivation [32,33] and (ii) in some cases, germline mutations within the coding region of telomerase, which impair the enzymatic activity of telomerase in proliferating hepatocytes (see Section 5) [34,35].

Reactivation of telomerase activity has been shown in more than 80% of HCCs, which suggests that telomerase activation is a rate-limiting process for liver cancer formation [21,36,37]. The reactivation of telomerase correlates with the upregulation of both essential components TERT and TERC, respectively. It has been reported that the re-expression of TERT and activation of telomerase occurs at early premalignant stages in regenerative nodules and cirrhotic livers [31,38,39]. Importantly, telomerase activity was detected both in HCC and in iCCA to a similar extent (80–85%) [36,40]. Thus, it is important to emphasize at this point that the majority of both HCCs and iCCAs are similarly telomerase-positive, highlighting the necessity of telomerase activity for telomere functionality and tumor progression.

Irrespective of the mechanism, insufficient telomerase activity leads to accelerated telomere shortening in proliferating liver cells and as a result to genomic instability by breakage–fusion–bridge cycles (see review by Meena et al. [41]). In cells with intact DNA-damage response (DDR) checkpoints, telomere shortening leads to senescence/apoptosis and functions as a tumor suppressor mechanism. On the other hand, in cells lacking functional DDR, telomere shortening promotes genome instability and tumor formation.

Consequently, telomere shortening is an important risk factor for tumor initiation in liver carcinogenesis. The risk of tumor formation drastically increases at the cirrhosis stage, which is characterized by increased hepatocyte senescence, and upon further cell division at the crisis checkpoint by apoptosis of hepatocytes. Several studies have shown that telomere shortening is more pronounced in liver carcinoma compared to the surrounding liver tissue (see review by Satyanarayana et al. [42]). Furthermore, the progressive shortening of telomeres and the inactivation of cell cycle checkpoints in premalignant lesions led to the identification of a preneoplastic-sequence in human hepatocarcinogenesis, suggesting that small cell changes (SCC) are more advanced precursor lesions compared to large cell changes (LCC) [43]. In addition, telomere shortening was more pronounced in HCCs with a high degree of aneuploidy compared to diploid HCCs [44,45]. In fact, several studies provide evidence for a role of telomere shortening in the induction of chromosomal instability and increased risk for tumor formation [46,47,48,49]. The importance of telomere shortening and dysfunctional telomeres in HCC initiation was shown in transgenic mouse models (see Section 3).

## 3. Mouse Models of Telomere Dysfunction in Hepatocarcinogenesis

To understand the severe situation of dysfunctional telomeres and telomere shortening during chronic liver disease, transgenic mouse models were used to analyze the functions of telomeres. To this end, it is important to note that, firstly, there is a substantial difference in the regulation of telomerase between mouse and human liver. Telomerase activity is detectable in resting mouse liver but not in resting human liver [30,50,51,52,53]. The limiting component, restricting telomerase activity in human tissues, is the catalytic subunit of the telomerase TERT [54]. Concordantly, there is a marked difference in *TERT* mRNA levels in human and mouse livers [52,53,54]. In fact, in vivo experimental evidence supports the idea that the species-specific differential regulation is based on different promoter organization [30,53,55]. Secondly, the average telomere length in laboratory mice is about five times longer than that of human telomeres, to some extent due to constitutive telomerase activity in mouse cells [56,57].

The telomerase knockout mouse (*Terc−/−*) lacking the RNA component of the telomerase enzyme was used to analyze telomere shortening in liver regeneration, chronic liver disease, and hepatocarcinogenesis [24,58,59,60]. Mice are characterized by the existence of longer telomeres compared to humans [56]. Mice of different backgrounds differ a lot in telomere length [61]. For this reason, the *Terc−/−* mouse has to be crossed until the third to the sixth generation, depending on the used background strain to generate mice with critically short telomere lengths [58,60,62]. In an experimental model of liver regeneration involving the removal of two-thirds of the liver by partial hepatectomy of G3 *Terc−/−* and *Terc+/+* mice, telomere shortening was observed to be a heterogeneous event at the cellular level, which led to the inhibition of a subpopulation of cells with critically short telomeres to enter the cell cycle and prevent those cells from participating in liver regeneration [58]. By comparing the mean telomere fluorescence intensities measured by FISH analysis, Satyanarayana and colleagues [58] observed no significant differences in *TERC+/+* mice between BrdU-positive cells (952.53 ± 144.19) and BrdU-negative cells (957.44 ± 130.57) but saw a dramatic reduction of the mean telomere fluorescence intensity of BrdU-negative cells (364.94 ± 116.45) in comparison to BrdU-positive cells (509.65 ± 101.30) in G3 *TERC−/−* mice. In a mouse model of experimentally induced acute liver damage in which *Terc−/−* mice were subjected to genetic, surgical and chemical impairment of the liver, dysfunctional telomeres were associated with defective liver regeneration and accelerated formation of liver cirrhosis, which could be partly rescued by adenoviral delivery of the telomerase RNA [24]. In an approach comprising three different cancer-prone model systems—(1) treatment with CCl_4_ (carbon tetrachloride), (2) treatment with DEN (diethylnitrosamine) and (3) a genetic model (urokinase plasminogen activator transgenic mice) in *Terc−/−* mice—it could be shown that telomere dysfunction has a differential impact on tumor initiation and tumor progression. In all three model systems, dysfunctional telomeres were associated with higher amounts of tumor initiation and a decline in tumor progression [63]. In mouse models of chronic liver damage achieved by crossing HBsAg-expressing mice (the mice express the hepatitis B surface antigen under the liver-specific albumin promoter [64]) with *Terc−/−* mice, contrary effects of telomere shortening were shown between the beneficial effect on suppression of tumor growth and the negative effect on organismal survival [59]. In another mouse model of chronic liver disease, HBsAg mice were crossed with *Terc−/−* and *Trp53* cKO mice. We generated mice with critically short telomeres by an intercross of *Terc−/−* and *Terc+/−* to generate siblings with loss of telomerase function in one group and telomerase expression in the other group. This study yielded the evidence for telomerase to be a critical component in the progression of *Trp53*-deficient hepatocellular carcinoma with short telomeres in the setting of chronic liver damage [60]. In addition, it was also shown that telomerase limits the accumulation of telomere dysfunction and the generation of aneuploidy by the activation of TRP53-independent checkpoints which suppress carcinogenesis [60]. Increased rates of chromosomal aberrations could be also shown in a DEN-induced liver cancer mouse model with dysfunctional telomeres. Telomerase knockout mice (*Terc−/−*) with chronic telomere dysfunction as well as a model of transient telomere dysfunction by inducing a dominant-negative variant of the TRF2 (telomeric repeat-binding factor 2) protein exhibited higher levels of chromosomal aberrations. In summary, the model of transient telomere dysfunction promotes chromosomal instability and liver carcinogenesis in telomerase-competent mice [65]. RAP1 (Ras-proximate-1 or Ras-related protein 1), like TRF2, is a component of the shelterin complex, which caps the telomere end for the protection of chromosome ends [66]. A recent publication suggested an important role of RAP1 in the protection of liver damage and liver carcinogenesis. DEN-induced *Rap1−/−* female mice were more prone to liver damage and hepatocellular carcinoma [67]. These models reflect the complexity and opposing roles of dysfunctional telomeres in hepatocarcinogenesis.

## 4. Telomere Shortening in Cholangiocarcinoma

Intrahepatic cholangiocarcinoma (iCCA) is the second most common malignant liver tumor which arises from the biliary tract and is characterized by a very poor prognosis with rising incidence and mortality in recent years [68,69]. The main risk factors described for HCC are also reported for iCCA. Additional risk factors are primary sclerosing cholangitis (PSC), hepatobiliary flukes, biliary duct cysts and hepatolithiasis [13]. A study by Verma and colleagues analyzed telomere shortening during aging in normal liver with no history of liver disease. Interestingly, they observed that the cholangiocytes exhibited the longest telomeres compared to all other analyzed intrahepatic lineages [70]. Similar to CD4^+^ and CD8^+^ lymphocytes, no significant telomere shortening was observed in cholangiocytes and hepatocytes of individuals without liver disease during aging. The authors only observed an age-related telomere shortening in Kupffer cells and stellate cells [70]. On the other hand, a consistent telomere shortening was reported during the development of biliary tract carcinoma, starting early in carcinogenesis in the inflamed biliary tract, metaplasia, dysplasia and carcinoma [71]. In contrast, the normal and the inflamed epithelium of the biliary tract showed a uniform telomere length [71]. Within cholangiocarcinoma, a frequent intratumoral heterogeneity of telomere length is reported [71]. As indicated above, telomere shortening in hepatocytes triggers cellular senescence in the context of intact DDR checkpoints. Similarly, an investigation of telomere shortening and senescence in the pathogenesis of primary biliary cirrhosis (PBC) showed telomere shortening. Moreover, DNA damage accumulation was detectable in biliary epithelial cells in the damaged small bile ducts and bile ductules in PBC in comparison to normal-looking bile ducts and bile ductules in PBC, chronic viral hepatitis and normal livers [72]. Of note, the accumulation of DNA damage foci correlated with increased expression of p16^INK4a^ and p21^WAF1/Cip1^, which characterize biliary cellular senescence [72].

## 5. Loss of Function Mutations in Telomerase Components

Germline loss-of-function mutations in the telomerase components were found in a variety of human diseases, including dyskeratosis congenita, aplastic anemia, familial idiopathic fibrosis and acute myeloid leukemia [73,74,75,76,77,78,79,80]. These mutations provoked an impaired tissue regeneration due to telomere dysfunction and stem/progenitor cell exhaustion. Similar mutations were also reported in a subset of liver cancer samples [34,35]. The authors analyzed *TERT* and *TERC* mutations in buccal mucosa tissue and peripheral blood of patients with liver cirrhosis and compared them with healthy non-cirrhotic controls. An increased number of telomerase mutations were found in the group with liver cirrhosis. The study by Calado et al. [34] reported nine patients with a mutation in the *TERT* gene and one patient with a mutation in the *TERC* gene among 134 patients with liver cirrhosis. Similarly, Hartmann et al. [35] reported mutations in the *TERT* and *TERC* genes in 16 out of 521 patients. The Calado study reported a significantly higher allele frequency for the gene variants in the *TERT* and *TERC* genes in patients with cirrhosis (allele frequency 0.037) compared to controls (0.008; *p* = 0.0011). A similar result was shown by the Hartmann study, which stated an increased incidence of telomerase mutations detected in cirrhosis patients (allele frequency 0.017) compared to non-cirrhotic controls (0.003, *p* = 0.0007). The mutations in telomerase components led to decreased telomerase activity in comparison to wildtype telomerase enzyme activity. Consequently, patients with these mutations showed shorter telomeres in peripheral white blood cells [34,35]. Rare *TERT* mutations were also reported in patients with nonalcoholic fatty liver disease (NAFLD). Here, an enrichment of *TERT* mutations could be found in NAFLD-associated HCC [81]. Functional evaluation of these mutations exposed reduced protein synthesis from some of the mutations compared to the TERT wild-type protein. It is speculated that these *TERT* mutations could also impair the DNA-binding function of TERT. In summary, these results indicate that *TERT* mutations result in impaired telomerase activity, accelerated telomere shortening and impaired regeneration in chronic liver disease. These findings are supported by the above-mentioned studies indicating low/absent telomerase activity in resting liver and telomerase activation in the regenerating liver [30,31]. Taken together, these studies show that telomerase activity acts as a protective mechanism in chronic diseases to prevent telomere shortening during accelerated cell proliferation, whereas *TERT* mutations result in telomere shortening and may promote hepatocarcinogenesis by dysfunctional telomeres.

## 6. Telomerase Reactivation during Hepatocarcinogenesis

It has been proposed for a long time that up to 90% of human tumors can reactivate telomerase [20,82]. Telomerase reactivation is associated with the alteration of transcriptional regulators of the *TERT* promoter in cancer, *TERT* promoter mutations or rearrangements and DNA copy number amplifications [30,83,84,85,86,87,88]. Reactivation of the telomerase enzyme has been shown in more than 80% of HCCs, which suggests reactivation of telomerase as a rate-limiting process for liver cancer formation. The reactivation of telomerase correlates with the upregulation of both essential components, *TERT* and *TERC*. It has been reported that the re-expression of *TERT* and re-activation of telomerase occurs at early premalignant stages in regenerative nodules and cirrhotic livers [31,38,39]. Telomerase activation was also reported in iCCA (85%) [40]. Thus, it is important to note at this point that the majority of HCCs and iCCAs are telomerase-positive, highlighting the necessity of telomerase activity for telomere functionality and tumor progression in the two most common liver cancer entities.

### 6.1. TERT Promoter Mutations

*TERT* promoter mutations which result in increased *TERT* expression were first identified in melanoma and were subsequently reported in other cancers like bladder cancer, glioma, thyroid cancer and HCC [84,89,90,91]. In the following, we present data that report on *TERT* promoter mutations in HCC, iCCA and tumors with a mixed-differentiation HCC/iCCA (Table 1, Table 2 and Table 3). These data were achieved by PubMed literature search, using the keywords “*TERT* promoter mutations”, “liver carcinoma” and “biliary tract cancer”. Additionally, the list includes studies that analyzed *TERT* promoter mutations in different tumor entities. Due to limited sample sizes in the reported studies, we excluded studies/data on rare entities, such as fibrolamellar carcinoma (FLC) and cholangiolocellular carcinoma (coCC), as well as studies/data based on liquid biopsies. In HCCs, *TERT* promoter mutations were identified with an overall prevalence of 20–82% as the most frequent somatic genetic alterations (Table 1) [92,93,94,95,96,97,98,99,100,101,102,103,104,105,106,107,108,109,110,111,112,113,114,115,116,117].

However, it should be noted that in 80% of cases which lacked *TERT* promoter, mutations showed an enhanced *TERT* expression. Moreover, telomerase activity was detectable, indicating the existence of alternative mechanisms of telomerase reactivation during liver carcinogenesis [118]. For example, it was shown that the insertion of HBV in the *TERT* promoter region can activate *TERT* gene expression and telomerase promoting hepatocarcinogenesis in HBV-related HCC [119,120]. Unlike HBV, HCV is not able to integrate into the host genome, but HCV can also induce chromosomal instability by direct effects of its proteins [121]. Interestingly, mutations in the *TERT* promoter were identified to be significantly more common in HCV-related HCC tumors compared with tumors without HCV infection [93]. It is also notable that the *TERT* promoter mutation frequency is higher in HCV-related HCC (64%) compared to HBV-related HCC tumors (37%) [98].

The occurrence of *TERT* promoter mutations in liver carcinogenesis is identified in premalignant lesions, and the prevalence of mutations gradually increased with the degree of dysplasia, indicating that *TERT* promoter mutations are highly associated with the stepwise transformation from premalignant dysplastic nodules to malignant HCC. *TERT* promoter mutations were identified in 6% of low-grade dysplastic nodules, 19% of high-grade dysplastic nodules and 61% of early HCCs. Interestingly, *TERT* promoter mutations were not detected in cirrhotic liver [94,117]. *TERT* promoter mutations are so far the earliest recurrent genetic events in cirrhotic preneoplastic lesions and belong to the most frequent alterations in hepatocellular carcinoma [94,122]. These results support the idea that telomerase reactivation is required for the malignant transformation of liver cells from cirrhosis to cancer.

**Figure 1:**
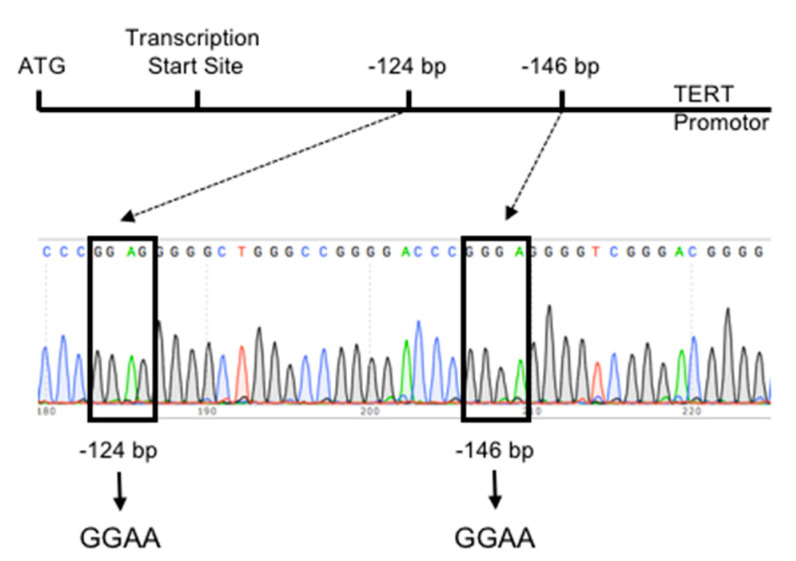
Identification of *TERT* promoter mutations in HCCs. Two hotspot mutations in the *TERT* promoter region generate de novo E-twenty-six (ETS) binding sites for transcription factors of the ETS family by exchange of nucleotides.

In HCC, *TERT* promoter mutations at several positions were described. The two most frequent mutations occur at positions −124 (G > A) and −146 (G > A) upstream of the ATG translation start site (Figure 1) [98,106]. Interestingly, the *TERT* promoter mutation at the −124 bp hotspot appears more often compared with the *TERT* promoter mutation at the −146 bp hotspot [93,94,96,97,98,100,101,106,108,111,112,114] was shown that the *TERT* promoter mutations generate a de novo consensus binding site for the E-twenty-six (ETS) transcription factor family [83,84], leading to an increase in TERT protein amounts, telomerase activity and telomere length [123]. Recently a new *TERT* promoter mutation was found in 7.5% of the analyzed HCCs at the position −297 (C > T) upstream of the ATG translation start site, which creates an AP2 consensus sequence [104].

Interestingly, the *TERT* promoter mutation frequency in HCC differs geographically. By comparing different studies, Pezzuto et al. [106] reported that *TERT* promoter mutations seem to be most common in Europe (56.6%). In Africa, *TERT* promoter mutations were identified in 53.3% and in Asia in 42.5% of HCCs. The overall *TERT* promoter mutation rate in America is around 40% [106].

In contrast to frequent mutations in the *TERT* promoter region in HCC, *TERT* promoter mutations are less frequently analyzed in cholangiocarcinoma [92,95,96,106,116,124,125,126]. These studies identified *TERT* promoter mutations in 31–47% of the HCCs. Nakamura et al. analyzed the largest dataset of 145 iCCAs (named as ICCs in the study). A *TERT* promoter mutation was detected only in one iCCA sample. Furthermore, the same study also analyzed a set of 86 extrahepatic cholangiocarcinomas, with none of them exhibiting a *TERT* promoter mutation [125].

While no *TERT* promoter mutation was detected in iCCA samples in the majority of studies [92,95,96,116], in some studies, performed on lower sample numbers, a low frequency of *TERT* promoter mutations were observed in iCCA [106,124,126] (Table 2).

The study by Fujimoto et al. was focused on different subtypes of liver cancer displaying biliary phenotype (LCB). They described *TERT* promoter mutations in 5.12% (4/78) of intrahepatic cholangiocarcinoma and in 53.3% (8/15) of combined hepatocellular cholangiocarcinoma [124].

The high percentage of *TERT* promoter mutations in HCC/iCCA may reflect the presence of the HCC part consisting of the *TERT* promoter mutation or indicate that both tumor types may arise from the same cell type of origin (Table 3).

**Figure 2:**
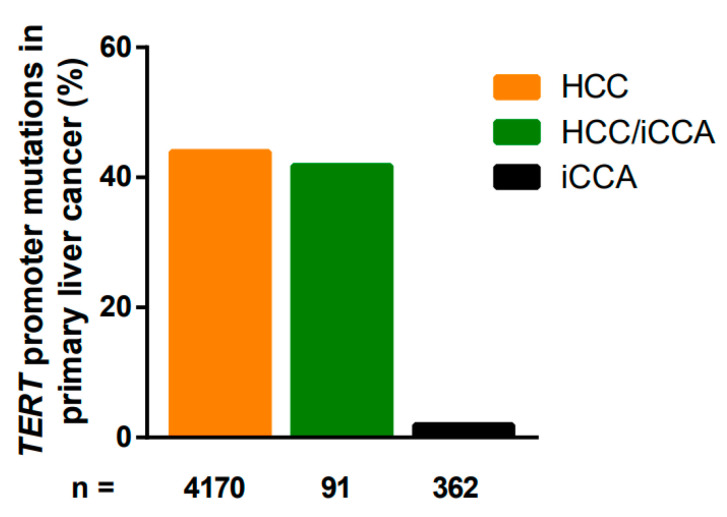
Distribution of *TERT* promoter mutations in liver carcinoma.

In addition, a clear difference between hepatitis-positive LCBs and hepatitis-negative LCBs was observed (20% vs. 6%) [124]. Taken together, the fact that *TERT* promoter mutations are detectable only in a small subset of cholangiocarcinoma indicates that the mechanism of telomerase activation is evidently different from telomerase activation in many HCCs (Figure 2).

### 6.2. Amplification and Genomic Rearrangements of TERT

Structural and numerical aberrations in the organization of the genome can lead to *TERT* promoter activation and eventually to telomerase reactivation. Frequent amplifications of the *TERT* gene have been shown in human cancer cell lines and in human tumors [85,128]. The amplified *TERT* gene was found in around 22% of hepatocellular carcinoma and shows a higher incidence in poorly differentiated hepatocellular carcinoma [129]. Nevertheless, the *TERT* mRNA level did not correlate with the number of *TERT* gene copies, as was also reported in colorectal cancer [129,130]. Focal amplifications of 5p15.33 (*TERT*) were observed with a reduced overall survival, independently of other clinicopathological parameters in patients with hepatocellular carcinoma [131]. However, it should be noted that *TERT* amplifications do not always lead to increased *TERT* mRNA levels, which are by themselves not assignable to increased TERT translation levels.

Structural rearrangements in the *TERT* gene have been shown in several cancers like neuroblastoma, renal cell carcinoma, sarcoma and prostate carcinoma, and also in liver carcinoma [82,86,132,133]. A specific event that occurs in hepatocellular carcinoma is integration of viral genomes. Integration of the Hepatitis B virus into the host genome has been described in hepatocellular carcinoma, frequently in the *TERT* promoter region [98,99,120,134,135,136]. The high frequency of HBV integration within the *TERT* promoter region might be a reason for a low rate of *TERT* promoter mutations in HBV-related HCC, which is known to induce telomerase transcription [94,119]. This could represent an alternative mechanism or two different mechanisms of telomerase reactivation.

AAV2 (adeno-associated virus type 2) is the second virus reported to integrate into HCC cell lines and hepatocellular carcinoma of human patients [137]. The insertion of AAV2 can take place in the *TERT* promoter region and thereby lead to overexpression of the *TERT* gene [137]. The AAV2 genome achieves a liver-specific enhancer-promoter activity in the 3′UTR and binding sites of hepatic transcription factors, which might be linked to *TERT* overexpression and telomerase reactivation [138].

### 6.3. Altered Transcriptional Regulation of TERT Gene Expression

Studies addressing telomerase activation in liver cells mainly focused on *TERT* gene regulation in hepatocytes and HCC. The RB/E2F gene regulatory circuit regulates *TERT* promoter activity during liver regeneration and cancer [30]. The role of the RB/E2F circuit in *TERT* regulation is supported by a recent report showing that the Human Krüppel related 3 (HKR3) is capable of repressing *TERT* gene expression in HCC cell lines, resulting in the subsequent activation of CDKN2A (encoding the p16 tumor suppressor factor) and cell cycle inhibition as a tumor suppressor mechanism [139]. In another study, the RNA-binding fox-1 homolog 3 (RBFOX3) protein cooperates with AP2β to activate *TERT* gene expression in HCC cell lines, indicating a role of telomerase activation in promotion of HCC [140]. Concurrently, impaired E2F1 binding to the *TERT* promoter has been shown to correlate with increased patient mortality [141].

On the other hand, the bromodomain PHD finger transcription factor (BPTF) seems to regulate *TERT* gene expression via nucleosome remodeling in HCC cell lines [142]. BPTF is required for c-MYC transcriptional activity in carcinogenesis, linking *TERT* gene activation to c-MYC, which was the first transcription factor shown to regulate *TERT* expression by direct promoter binding [143,144,145,146]. Of note, *c-MYC* amplification correlates with liver cancer progression, mainly HCC, and to a lesser extent, iCCA [147,148]. In a recent study using a genome-wide shRNA screening strategy in HepG2 cell line, Chr15orf55 (also known as NUTM1) and Chr7orf43, two regulatory factors with currently unknown functions, were found to activate *TERT* gene expression through SP1 or YAP1, respectively [149]. The exact nature of these factors and their role in HCC and iCCA remain yet to be clarified.

### 6.4. Epigenetic Mechanisms in the Regulation of TERT Gene Expression

There are a number of studies indicating that *TERT* promoter methylation and histone acetylation may be involved in the regulation of *TERT* transcription and hence telomerase activity. The mechanism of MAD1-mediated repression involves modification of the chromatin structure by histone deacetylation [146]. In fact, treatment of telomerase-negative human cell lines with the histone deacetylase inhibitor TSA (trichostatin A) results in the reactivation of *TERT* gene expression and telomerase activity [150], whereas the overexpression of *HDAC1* causes the repression of the *TERT* gene and telomerase activity [151]. To what extent histone acetylation/deacetylation is involved in *TERT* regulation during carcinogenesis remains pending.

The overexpression of DNMTs is thought to be responsible for suppressing the expression of tumor suppressor proteins by methylating the promoters of their genes, which leads to the early switching off of these factors. DNMT3B in particular is strongly overexpressed in breast tumors and correlates with the degree of tumorigenesis [152]. It can therefore also be assumed that the epigenetic regulation of *TERT* gene expression takes place indirectly, namely via the promoter methylation of genes for transcription factors which are responsible for *TERT* gene expression. A recent report showed the cooperation between TERT and the transcription factor SP2 to stimulate *DNMT3B* transcription, while *TERT* depletion inhibited *DNMT3B* expression [153]. Higher levels of *TERT* and *DNMT3B* expression predicted shorter survival in HCC patients based on the TCGA database [153].

On the other hand, the methylation status of the *TERT* promoter was studied in some detail. Initial reports regarding the methylation state and the activity of the *TERT* promoter were contradictorily unequivocal: while Devereux et al. [154] and Dessain et al. [155] did not observe an impact of promoter methylation of *TERT* expression, Guilleret et al. [156] found a correlation between the methylation state and the activity of the *TERT* promoter. Moreover, studies on *TERT* promoter methylation in HCC revealed contradictory results. Zhang et al. [157] observed that aberrant methylation of *TERT* promoter in HCC patients of the Han Chinese population showed a nearly 56-fold increase of *TERT* expression from the hypermethylated promoter. In contrast to that study, an analysis of 106 patient tissues (64 with HCC and 42 without liver disorders) and hepatocarcinoma cell lines revealed that the *TERT* promoter was methylated in normal liver but was hypomethylated in most of the hepatocellular carcinomas [158].

Recent studies point out the importance of a comprehensive mapping of the methylation landscape within the *TERT* promoter using more advanced technologies, arguing that the methylation analysis of a small number of CpG sites may not represent the methylation landscape of the whole *TERT* promoter [159]. Using a next-generation sequencing technique, the authors performed a comprehensive methylation analysis and revealed a *TERT* hypermethylated oncological region (THOR) defining a 433 bp genomic region within the *TERT* promoter as a cancer-associated epigenetic mechanism of *TERT* upregulation. This region encompasses 52 CpG sites and is located upstream of the *TERT* promoter mutations (TPMs) [159]. Five CpG sites within THOR accurately presented the average THOR methylation and were used for a bigger screen of 1352 tumor samples and 80 normal samples. Of the tumor samples 91.4% exceeded the median THOR methylation level detected in normal samples. Low THOR methylation levels were detected in thyroid cancers, which was linked to a lower malignant potential and a better prognosis [159]. Tumors from skin and bone showed low and heterogeneous methylation levels, which indicates the use of other mechanisms of telomere maintenance like *TERT* promoter mutations or the alternative lengthening of telomeres (ALT). In line with these results, Esopi et al. [160] described hypermethylation in the region upstream of the recurrent C228T and C250T promoter mutations in immortalized and cancer cell lines (including hepatocarcinoma cell lines). In contrast, non-malignant primary cells were rather hypomethylated [160]. Interestingly, the authors could show on an allele level that the hypermethylation of *TERT* promoter sequences in cancer cells is associated with *TERT* repression, while the remaining unmethylated allele marked with an open chromatin is largely responsible for the *TERT* expression in cancer cells [160]. In cancers with *TERT* promoter mutations, the expressed allele is mutated, while the WT allele is silenced [160,161]. In summary, current data support the assumption that *TERT* promoter activity can be regulated by epigenetic mechanisms in a tissue- and cancer-type-specific manner.

## 7. Telomere and Telomerase-Based Cancer Therapy

The fact that telomerase is repressed in somatic tissues but active in cancer cells qualifies it as an ideal target for cancer therapy. On the other hand, impairment of telomere length and/or structure could be a target for tumor therapy as telomere length in cancer cells is usually shorter than in the corresponding normal tissue cells. Moreover, due to their high turnover, functional telomeres have to be rebuilt more often in tumor cells than in normal cells. Thus, deterioration of the intact telomere structure by compounds may represent an attractive target to cure cancer.

Several telomerase inhibitors have been used in preclinical studies and clinical trials, involving telomerase inhibitor molecules, such as the antisense oligonucleotide Imetelstat (GRN163L) or the small-molecule inhibitor BIBR1532, as well as the G-quadruplex stabilizers (BRACO, RHPS4, Telomestatin). There are also interesting alternative strategies such as telomerase vaccination (peptide-based: GV1001; mRNA-based: GRNVAC1) to induce anti-tumor responses or tumor cell lysis through *TERT* promoter-driven oncolytic adenovirus (Telomelysin (OBP-301)) or *TERT* promoter-driven pro-apoptotic protein (e.g., TRAIL) to take advantage of high telomerase activity in cancer cells for therapy options. For further reading, we refer to a recent excellent article, which discusses this topic in greater detail [162]. Furthermore, we refer to a recent review article, which summarizes clinical trials on telomerase-based cancer therapeutics [163].

## 8. Conclusions

The biology of telomere maintenance plays a major role in the process of cirrhosis formation as well as in initiation and progression in liver carcinoma. A shortening of telomeres occurs due to progressive chronic liver diseases and highly correlates with an increased tumor risk. Telomere shortening occurs in HCC as well as in iCCA. In addition, telomerase reactivation is detectable at a high frequency in both tumor entities. The mechanisms of telomerase activation seem, at least in part, to be different between the two liver cancer entities. *TERT* promoter mutations were more frequently observed in HCCs compared to iCCAs. The low frequency of *TERT* promoter mutations reported in iCCA might be due to a not-detected HCCs part (mixed differentiation) of the analyzed tumors. This links to the phenomenon that some tumor entities lacking specific mutations in the *TERT* promoter region and where other mechanisms leading to telomerase activation exist. As described above, the *TERT* promoter region contains binding sites for many transcription factors, i.e., c-MYC, E2Fs, and others, which contribute to the tissue-specific regulation of *TERT* gene expression [22]. It would be interesting to explore whether *TERT* expression is regulated differentially in HCC and iCCA. The identification of the regulatory mechanisms contributing to telomerase reactivation in HCC and iCCA could shed light on the differential tumor initiation and progression pathways and may provide alternative and specific therapy options.

**Figure 3:**
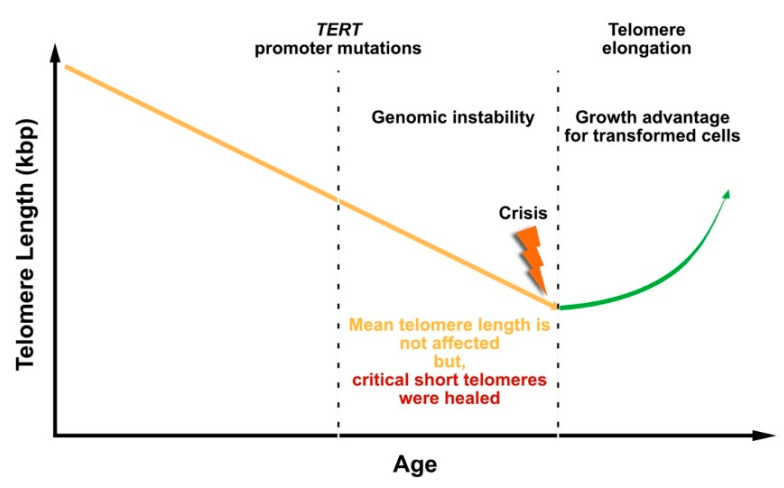
*TERT* promoter mutations are frequently involved in carcinogenesis and represent one way of telomerase activation.

The observation that *TERT* promoter mutations occur early during liver carcinogenesis highlights the importance of telomerase activity for tumor cell survival. Two possible scenarios are conceivable how telomerase contributes to tumorigenesis in liver cancer. On the one side, telomerase reactivation before entering the crisis checkpoint may stabilize critically short telomeres, providing growth advantage for cells with oncogenic mutations (Figure 3). In melanoma, a two-step mechanism was described, which showed that mutations in the TERT promoter region contribute to tumorigenesis [164]. This study showed that TERT promoter mutations are not sufficient to counteract telomere shortening, but they contribute to tumorigenesis by promoting the immortalization and chromosomal instability in two phases: (1) extend the cellular life span by healing the shortest telomeres without prevention of bulk telomere shortening, and (2) the existence of critically short telomeres conducts to genome instability and thereby, telomerase is further up-regulated to proceed cell proliferation.

On the other side, early reactivation of telomerase may be related to its non-canonical functions. There is strong experimental evidence indicating that oncogenic mutations or mutations which result in genome instability induce cellular senescence by inducing telomere replication stress [41,165]. This mechanism functions to limit continuous proliferation of cells carrying detrimental mutations, preventing tumorigenesis.

However, telomerase activity can alleviate telomere replication stress by a yet-unidentified mechanism and promote tumorigenesis [22,88,166,167].

Whether telomere maintenance or even elongation of telomeres during chronic liver diseases or an inactivation of the telomerase could be beneficial for the treatment of liver carcinoma remains to be elucidated in future studies.

## Figures and Tables

**Table 1:** *TERT* promoter mutations in hepatocellular carcinoma.

Tumor Type	Number of Samples	*TERT* Promoter Mutation	Etiology		Reference
			−124 bp	−146 bp	
HCC *	61	44.2% (27/61)	HCV		Killela et al., 2013 [96]
			62.5% (10/16)	0 (0/16)	
			HBV		
			26.6% (4/15)	0 (0/15)	
			ETOH		
			100% (2/2)	0	
			cyptogenic liver disease		
			100% (1/1)	0	
			unknown		
			50% (8/16)	0 (0/16)	
HCC	70 ^#^	71% (50/70)	HCV		Chianchiano et al., 2018 [97]
			87.5% (35/40)	2.5% (1/40)	
			HBV		
			0 (0/7)	0 (0/7)	
			ETOH		
			16.6% (1/6)	0 (0/6)	
			HCV/HBV		
			100% (2/2)	0	
			unknown		
			73.3% (11/15)	0 (0/15)	
HCC	457	54.2% (248/457)	HCV		Totoki et al., 2014 [98]
			62.2% (117/188)	1.6% (3/188)	
			HBV		
			28.7% (31/108)	3.7% (4/108)	
			HCV/HBV		
			66.6% (8/12)	0 (0/12)	
			NBNC ^†^		
			53.6% (80/149)	3.3% (5/149)	
HCC	104	65% ^‡^ (68/104)	HCV		Kawai-Kitahata et al., 2016 [99]
			80% (40/50)		
			HBV		
			32% (9/28)		
			ETOH		
			83% (10/12)		
			unknown		
			64% (9/14)		
HCC	160	28.8% ^§^ (46/160)	HCV		Lee et al., 2017 [100]
			60% (3/5)		
			HBV		
			32.7% (19/58)		
			ETOH		
			28.5% (6/21)		
			others		
			23.6% (18/76)		
HCC	105	39% (41/105)	HCV		Lee et al., 2016 [93]
			83.3% (5/6)		
			HBV		
			29.4% (23/78)		
			ETOH		
			37.5% (3/8)		
			unknown		
			76.9% (10/13)		
HCC	44	34% (15/44)	HBV		Cevik et al., 2015 [101]
			13% (3/23)	13% (3/23)	
			unknown		
			33.3% (7/21)	9.5% (2/21)	
HCC	97	54.6% (53/97)	HCV		Kwa et al., 2020 [102]
			71% (22/31)		
			HBV		
			36.4% (8/22)		
			NBNC		
			52.3% (23/44)		
HCC	10	50% (5/10)	HCV		Rudini et al., 2018 [103]
			42.9% (3/7)	14.3% (1/7)	
			ETOH		
			0 (0/2)	0 (0/2)	
			unknown		
			100% (1/1)	0 (0/1)	
HCC	67	43.3% (29/67)	ESC-NA		Lombardo et al., 2020 [104]
HCC	14	21.4% (3/14)	ESC-NA		Jospeh et al., 2019 [105]
HCC	127	50.4% (64/127)	HCV		Pezzuto et al., 2016 [106]
			53.6% (59/110)		
			HBV		
			41.7% (5/12)		
			NBNC		
			0 (0/5)		
HCC	11	81.9% (9/11)	NAFLD		Kim et al., 2016 [107]
			100% (9/9)	0 (0/9)	
HCC	190	30% (57/190)	HBV		Yuan et al., 2017 [108]
			32.7% (50/153)		
			unknown		
			18.9% (7/37)		
HCC	375	20.3% (76/375)	classical HCC		Pilati et al., 2014 [109]
			54% (68/125)		
			HCC derived from adenomas		
			56% (5/9)		
			borderline lesions HCA/HCC		
			17% (3/18)		
			classical adenomas		
			0 (0/223)		
HCC	196	44.4% (87/196)	HCV		The Cancer Genome Atlas Research Network 2017 [110]
			61.3% (19/31)	3.2% (1/31)	
			HBV		
			22.5% (9/40)	2.5% (1/40)	
			HCV/HBV		
			50% (2/4)	0 (0/4)	
			NBNC		
			40.5% (49/121)	5% (6/121)	
HCC	88	29.6% (26/88)	low-grade dysplastic nodules		Nault et al., 2014 [117]
			6.3% (2/32)		
			high-grade dysplastic nodules		
			18.8% (3/16)		
			early HCC		
			60.9% (14/23)		
			progressed HCC		
			41.2% (7/17)		
HCC	276	31% (85/276)	HBV		Yang et al., 2016 [111]
			98.8% (84/85)	1.2% (1/85)	
non-clear cell HCC	259	33.2% (86/259)	94.2% (81/86)	5.8% (5/86)	Huang et al., 2017 [112]
clear cell HCC	57	26.3% (15/57)	100% (15/15)	0 (0/15)	
HCC	322	64.5% (208/322)	combined etiology		Calderaro et al., 2017 [113]
			64.5% (208/322)		
HCC	195	29.2% (57/195)	94.7% (54/57)	5.3% (3/57)	Chen et al., 2014 [114]
HCC	235	60.4% (142/235)	combined etiology		Schulze et al., 2015 [115]
			60.4% (142/235)		
HCC	35 **	31.4% (11/35)	81.8% (9/11)	18.2% (2/11)	Huang et al., 2015 [92]
HCC	78 **	47% (37/78)	100% (37/37)	0 (0/37)	Quaas et al., 2014 [95]
HCC	305 **	58.6% (179/305)	93.8% (168/179)	6.1% (11/179)	Nault et al., 2013 [94]
HCC	162 **	45% (73/162)	NA	NA	Barthel et al., 2017 [82]
HCC (K19−)	44 ***	59% (26/44)	100% (26/26)	0	Akita et al., 2019 [116]
HCC (K19+)	26 ***	31% (8/26)	100% (8/8)	0	
Total	4170	43.9% (1831/4170)			

* Only for hepatocellular carcinoma (HCC) patients with known clinical information; NA = data not available; ^#^ tumors from 24 patients; ^†^ no hepatitis infection; ^‡^ 66/68 tumors show mutation at −124 bp, and 2/68 patients show mutation at −146 bp; ^§^ 32/46 tumors show mutation at −124 bp, and 14/46 tumors show mutation at −146 bp; ** etiology was not described; *** etiology was not correlated; HBV: Hepatitis B virus; HCV: Hepatitis C virus; ETOH: Alcohol; HCV/HBV: Hepatitis c and Hepatitis C virus; ASH: Alcoholic steatohepatitis; NASH: Non-alcoholic steatohepatitis; PBC: Primary biliary cholangitis; PSC: Primary sclerosing cholangitis; NBNC: No hepatitis virus infected; ESC-NA: Etiology-specific classification is not available; NAFLD: non-alcoholic fatty liver disease.

**Table 2:** *TERT* promoter mutations in intrahepatic cholangiocarcinoma (iCCA).

Tumor Type	Number of Samples	*TERT* Promoter Mutation	Etiology		Reference
			−124 bp	−146 bp	
iCCA	145	0.70% (1/145)	HCV		Nakamura et al., 2015 [125]
			0 (0/10)	0 (0/10)	
			HBV		
			0 (0/7)	0 (0/7)	
			NBNC ^†^		
			0.8% (1/122)	0 (0/122)	
			unknown		
			0 (0/6)	0 (0/6)	
iCCA	78	5.12% (4/78)	HCV		Fujimoto et al., 2015 [124]
			22.2% (2/9)		
			HBV		
			9% (1/11)		
			NBNC ^†^		
			1.7% (1/58)		
iCCA	10	10% (1/10)	HCV		Joseph et al., 2019 [126]
			0 (0/5)		
			HBV		
			0 (0/2)		
			NASH		
			50% (1/2)		
			PBC		
			0 (0/1)		
CC	4	25% (1/4)	HCV		Pezzuto et al., 2016 [106]
			25% (1/4)		
iCCA	9 **	0 (0/9)	0 (0/9)	0 (0/9)	Huang et al., 2015 [92]
iCCA	52 **	0 (0/52)	0 (0/52)	0 (0/52)	Quaas et al., 2014 [95]
iCCA	28 **	0 (0/28)	0 (0/28)	0 (0/28)	Killela et al., 2013 [96]
S-iCCA	36 ***	0 (0/36)	0 (0/36)	0 (0/36)	Akita et al., 2019 [116]
Total	362	1.9% (7/362)			

^†^ No hepatitis infection; ** etiology was not described; *** etiology was not correlated.

**Table 3:** *TERT* promoter mutations in combined HCC/iCCA.

Tumor Type	Number of Samples	*TERT* Promoter Mutation	Etiology		Reference
			−124 bp	−146 bp	
cHCC/CC	15	53.3% (8/15)	HCV		Fujimoto et al., 2015 [124]
			83.3% (5/6)		
			HBV		
			0 (0/3)		
			NBNC *		
			50% (3/6)		
combined HCC-CC	20	70% (14/20)	HCV		Joseph et al., 2019 [126]
			81.8% (9/11)		
			HBV		
			0 (0/1)		
			HCV/HBV		
			100% (1/1)		
			ASH		
			100% (1/1)		
			NASH		
			100% (1/1)		
			ASH/NASH		
			0% (0/1)		
			PSC		
			100% (1/1)		
			unknown		
			33.3% (1/3)		
cHC-CC	53	30.2% (16/53)	HCV		Sasaki et al., 2017 [127]
			31.8% (7/22)		
			HBV		
			44.5% (4/9)		
			ETOH		
			40% (2/5)		
			NAFLD		
			0 (0/8)		
			unknown		
			33.4% (3/9)		
HCC-CC	3	0 (0/3)	HCV		Pezzuto et al., 2016 [106]
			0 (0/2)		
			HBV		
			0 (0/1)		
Total	91	41.8% (38/91)			

* No hepatitis infection.
